# Clarifying the Specific Role of Nurses in Rehabilitation is Key to Enhancing Interprofessional Collaboration: A Multi-method Study

**DOI:** 10.1097/RNJ.0000000000000538

**Published:** 2026-04-09

**Authors:** Cynthia Beigprégonne, Anja Pattschull, Charlotte Germain, Hadjira Bakiri, Christophe Luthy, Christophe Graf, Huguette Guisado, Celine Ventose, Gora Da Rocha Rodrigues

**Affiliations:** 1 Department of Medicine, Lausanne University Hospital, Lausanne, Switzerland; 2 Women-Mother-Child Department, Lausanne University Hospital, Lausanne, Switzerland; 3 Home Care Services, Les Soins Volants, Lausanne, Switzerland; 4 Department of Rehabilitation and Geriatrics, Division of General Medical Rehabilitation, Geneva University Hospitals, Geneva, Switzerland; 5 Department of Rehabilitation and Geriatrics, Division of Geriatrics and Rehabilitation, Geneva University Hospitals, Geneva, Switzerland; 6 Department of Rehabilitation, Geneva University Hospitals, Geneva, Switzerland; 7 Department of Rehabilitation and Geriatrics, Geneva University Hospitals, Geneva, Switzerland; 8 HESAV School of Health Sciences—Vaud, HES-SO University of Applied Sciences and Arts Western Switzerland, Lausanne, Switzerland

**Keywords:** Nurse’s role, rehabilitation nursing, leadership, interprofessional relations

## Abstract

**Purpose:**

This study aimed to clarify the perception of the specific nursing role among nurses and the interprofessional team in two rehabilitation units of a Swiss University hospital.

**Design and Methods:**

As part of an implementation project, we employed a multi-method design, including qualitative and quantitative approaches, to conduct a comprehensive contextual analysis in preparation for introducing an innovation in two rehabilitation units of the Geneva University Hospital. We performed 11 focus groups among nurses, nurse managers, other health care professionals, patients, and family members (*N*=50), analyzed using a six-stage thematic analysis approach. A structured questionnaire was additionally completed by 35 nurses.

**Results:**

The nursing role in rehabilitation was recognized but only partially deployed. Identified barriers included unclear role definitions, lack of leadership, organizational constraints and limited interprofessional understanding. Nurses prioritized patient autonomy and holistic care, while interprofessional teams primarily perceived nurses in delegated roles. Quantitative results highlighted the importance of prioritizing the nursing role but revealed challenges in interprofessional collaboration, particularly regarding feasibility and competence.

**Clinical Relevance to the Practice of Rehabilitation Nursing:**

Clarifying and reinforcing the contributions of rehabilitation nurses is essential to enhancing leadership, interprofessional collaboration, and patient outcomes. Recommended strategies include reflective practice, interprofessional training, and promoting nursing-specific competencies early in education (undergraduate/postgraduate training).

**Conclusion:**

Clarifying the specific nursing role in rehabilitation and participating in interprofessional training are key to improving collaboration and strengthening leadership. Future efforts should focus on integrating role clarification and leadership into nursing education to optimize teamwork and patient outcomes.

## Introduction

Interprofessional collaboration is the cornerstone of high-quality care in inpatient rehabilitation, ensuring a coordinated approach to patient recovery (Singh et al., 2018). By uniting diverse health care professionals, this collaboration supports patients in regaining independence, improving quality of life, and achieving social and professional reintegration. The Pan-Canadian Consortium for Interprofessionalism in Health highlights six key competencies for effective collaboration: communication, person-centered care, role clarification, teamwork, collaborative leadership, and conflict resolution (Canadian Interprofessional Health Collaborative, 2010). Among these, role clarity is critical in optimizing interprofessional teamwork and enhancing patient outcomes.

For rehabilitation nurses, role clarity is essential. When team members understand and respect their scope of practice, responsibilities are effectively distributed, and patient care is optimized (Registered Nurses’ Association of Ontario [RNAO], 2013). Conversely, unclear roles may limit recognition of rehabilitation nurses’ contributions, limiting their involvement in decision-making (Ehrlich et al., 2022; Hepp et al., 2015). This challenge is amplified when nurses struggle to assert themselves, leading to miscommunication and, ultimately, a decline in care quality (Hepp et al., 2015; Brault et al., 2014). Clearly articulating their role within the team is essential to ensuring their contributions are fully recognized.

Rehabilitation nurses play a central role as care coordinators, ensuring continuity and advocating for patients’ needs (Kirkevold, 2010; Tanlaka et al., 2023; Vaughn et al., 2016). Beyond medical issues, nurses’ interventions include targeted actions to support patients in performing tasks of daily living, with the goal of regaining functionality. They actively promote patient autonomy by fostering motivation, providing education on self-management strategies, and supporting psychosocial adaptation (Vaalburg et al., 2023; Pryor, 2010). This multifaceted role requires advanced skills in leadership, communication, and adaptability. Despite their essential contributions, they often face challenges in clearly defining and articulating their roles within the interprofessional care team (physician and physiotherapeutic roles), which may undermine their clinical leadership and effective role deployment (Tanlaka et al., 2023). To effectively communicate their role, they must first develop a comprehensive understanding of their own professional scope and gain confidence in the unique contributions they bring to the team (Canadian Interprofessional Health Collaborative, 2010P; RNAO, 2013). However, while the contributions of rehabilitation nurses have been previously described in specific contexts, how their role is perceived by nurses, other health professionals, patients, and families remains unclear. Current evidence remains mostly descriptive, leaving a limited understanding of how professional identity shapes role articulation in interprofessional teams. These gaps hinder the development of strategies to optimize and communicate the nursing role.

Closely tied to role clarity is professional identity, shaping how rehabilitation nurses perceive their roles within the health care system (Joseph et al., 2021; Rasmussen et al., 2018). It is shaped by norms, values, ethics, knowledge and leadership of the nursing discipline (Godfrey & Young, 2020; Joseph et al., 2021). Identity encompasses three dimensions: personal values (“Who I Am”), role definition (“What I Do”), and context (“Where I Do It”) (Rasmussen et al., 2018). A strong professional identity is reinforced when nurses clearly understand their contributions and can effectively communicate their role within the interprofessional team (Johnson et al., 2012; Joseph et al., 2021; Rasmussen et al., 2018). By reflecting on their values, skills and goals, rehabilitation nurses can strengthen their professional identity, enhance clinical leadership, and optimize their role within collaborative care settings, while engaging in dialogue with other professionals to ensure their roles are recognized and valued. Their specialized contributions are vital for fostering successful teamwork and sustainable patient outcomes. Hence, addressing gaps in role clarity and professional identity is key in consolidating the specific nursing role in rehabilitation.

Given the complexity of role perception in rehabilitation nursing, we employed a multi-method design to provide both depth and breadth of understanding. Qualitative focus groups with nurses, nurse managers, other health care professionals, patients, and families enabled in-depth exploration of perspectives and lived experiences regarding the role of rehabilitation nurses in the interprofessional environment, while a quantitative survey among nurses offered broader insights into the perceived importance, feasibility, and self-assessed competence related to rehabilitation nursing practice. Conducted in two rehabilitation units of a Swiss university hospital, we aimed to clarify the articulation of the nursing role and to identify gaps and opportunities, with the goal of informing evidence-based strategies to strengthen the integration and impact of rehabilitation nurses within interprofessional teams.

## Methods

We employed a multi-method design, with qualitative and quantitative components, conducted in parallel but analyzed independently, each providing complementary insights. This approach enabled a comprehensive contextual analysis in preparation for the introduction of an innovation (Figure 1). The study adhered to COREQ (Consolidated Criteria for Reporting Qualitative Research) (Tong et al., 2007) for the qualitative component and STROBE (Strengthening the Reporting of Observational Studies in Epidemiology) (Cuschieri, 2019) for the quantitative descriptive aspect. The project progressed through multiple stages, following the Integrated Promoting Action on Research Implementation in Health Services framework (I-PARIHS) (Harvey & Kitson, 2015). This article focuses on the context analysis conducted in 2020, guided by I-PARIHS. Master’s students in Nursing Sciences from the Institute of Higher Education and Research in Healthcare, affiliated with the University of Lausanne and Lausanne University Hospital, were engaged as part of an implementation project within the Master of Science in Nursing Sciences program (MScSI). They jointly assumed the roles of coordinators, facilitators, data collectors, data managers, and data analysts. In addition, a resource group composed of faculty members, nurse directors, nurse managers, and a secretary contributed by assuming coordination and facilitation responsibilities, while faculty members provided expert facilitation and ensured the validation of the analyses and results.

**Figure 1:**
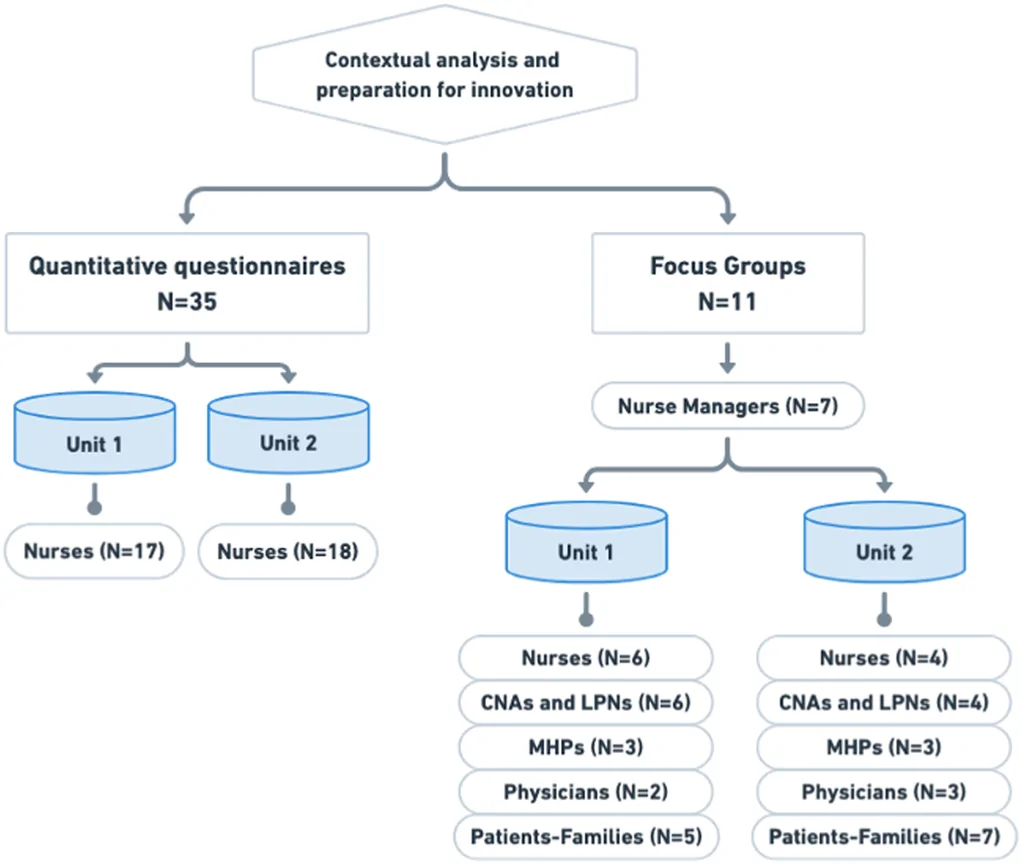
Methodological framework and study participants. This figure illustrates the study methodology, including the number of participants (*N*) in each category. A total of 11 focus groups were conducted, involving 50 participants, alongside quantitative questionnaires completed by 35 participants. CNAs=Certified Nursing Assistants; LPNs=Licensed Practical Nurses; MHPs=Multidisciplinary Healthcare Professionals.

The project was developed within the Rehabilitation Department of Geneva University Hospitals, a 700-bed tertiary care center that also serves as the primary health care facility for the region. The department is characterized by a median length of stay of 16 days and an average of 21 days. Most patients are discharged home, while 7–8% die during hospitalization, and 15–20% require permanent institutionalization. The project involved two rehabilitation units (Unit 1 and Unit 2), each with its own care context and organizational culture. Unit 1 is a traditional post-acute care rehabilitation unit of 54 beds, applying the Caring philosophy since 2015 (Guisado et al., 2019), focusing on the role of rehabilitation nurses, which improved nurse satisfaction (Roulin et al., 2020). The Caring philosophy emphasizes compassionate, holistic, patient-centered care that respects individual needs, promotes healing, and fosters meaningful connections between caregivers and patients (Watson et al., 1998). Within this framework, nurses have actively participated in interdisciplinary meetings for several years. Unit 2 is a post-acute care unit dedicated to general medical rehabilitation and psychosocial care, with a particular focus on comprehensive rehabilitation and multidisciplinary treatment. Unit 2 is part of the general medical rehabilitation division, a subacute internal medicine department with 96 beds.

### Theoretical Framework and Procedure

The I-PARIHS accelerates translating research into action through innovation, recipients, context, and facilitation across four iterative stages (Harvey & Kitson, 2015). During the first stage, a resource group composed of master’s students, faculty members, nurse directors, nurse managers, and a secretary identified and clarified factors that undermine research. The second stage involved context analysis and preparation for the introduction of the innovation, achieved through the focus groups and the quantitative questionnaire. The final stage implemented strategies through an action plan to promote adoption, adaptation, and sustainability of the innovation, with continuous evaluation, barriers analysis, and adjustments based on feedback and data. The third and fourth stages occurred later and lie beyond this study’s scope.

## Quantitative Design

### Study Participant

All nurses working in the two rehabilitation units were invited to complete the questionnaire between May and August 2021, and 35 nurses voluntarily participated on an opt-in basis (Figure 1). The inclusion criteria encompassed being a permanent health care worker in the involved units. Temporary health care workers and travel nurses were excluded.

### Data Collection

A quantitative questionnaire was designed in French by the four master’s students to assess the unique contributions of rehabilitation nurses (Supplemental Table 1, Supplemental Digital Content 1, http://links.lww.com/RNJ/A64). Specifically, nurse interventions, understanding of rehabilitation nursing, caring philosophy, and patient-family perceptions were evaluated based on the Association of Rehabilitation Nurses’ competency model, which defines four core competencies: leadership, interprofessional care, nurse interventions, and health promotion (Vaughn et al., 2016). Competencies were categorized by experience level into novice (0–2 years), intermediate (3–5 years), and expert (over 5 years) (Benner, 1984). The questionnaire included 17 Likert-scale items (1: *not at all* to 4: *very*) assessing the importance, competence, and feasibility of competencies, along with three open-ended questions on caring philosophy and qualitative insights. Socio-professional data were also collected. Before the study’s initiation and as part of a pilot phase, the questionnaire was pretested and refined based on feedback from nurses at an independent rehabilitation unit at Geneva University Hospital to ensure clarity, relevance, and appropriateness. The questionnaire’s internal consistency was evaluated using Cronbach’s alpha, yielding a coefficient of .96, indicating high reliability for research purposes. Following the pretesting phase, it was deployed online and distributed to all nurses in both study units via Forms©.

### Data and Statistical Analysis

Descriptive results were expressed as proportions (percentages) or medians with interquartile ranges (IQR). For the comparison of participant characteristics between Units 1 and 2, we performed Fisher’s exact test for categorical variables and the Wilcoxon rank-sum test for continuous variables. For comparisons of questionnaire scores across multiple groups, the Kruskal-Wallis, followed by Dunn’s test with multiple testing correction, was used to avoid assumptions of normality. All analyses were performed using the R software, version 4.2.2.

## Qualitative Design

### Study Participants

To gain a comprehensive understanding of the field’s complexity, we examined interprofessional teams and practices in each unit individually. A total of 11 focus groups were conducted with 50 participants in total, distributed across the groups. Each participant took part in only one focus group. Each focus group was composed of participants from a single professional category selected by their availability on the day of the focus group (nurse managers, nurses, Certified Nursing Assistants [CNAs], Licensed Practical Nurses [LPNs], Multidisciplinary Healthcare Professionals [MHPs], Physicians, patients/families). We chose focus group discussions over individual interviews to capture the interactions, collective reflections, and dynamics within and across professional groups, which were particularly relevant for exploring interprofessional collaboration. Patients and family members were recruited on a voluntary basis through a patient association. The association disseminated information about the study to its members, who were then invited to express their interest in participating in the focus group. The inclusion criteria encompassed being a permanent health care worker and being a patient or a family member of a patient who was either currently hospitalized or had previously been hospitalized for rehabilitation purposes.

### Data Collection

Eleven semi-structured focus groups were conducted to capture heterogeneity of experiences and perspectives between July and August 2021. Participants were intentionally recruited to represent a diverse range of roles and viewpoints, including nurses, nurse managers, members of the interprofessional team (CNAs/LPNs, MHPs, physicians), as well as patients and their families. Five focus groups, each involving six participants, were conducted per unit. These included one group with nurses, one with CNAs and LPNs, one with MHPs (including a physiotherapist, an occupational therapist, and a dietitian), one with physicians, and one with patients and families. Nurse managers from both units were convened into the last focus group. Each focus group lasted 60 minutes and was digitally recorded.

The aim of the focus groups was to explore: for nurses, the intention behind care, vision, and values of rehabilitation nursing through the four domains of the nursing metaparadigm: care, person, health, and environment (Fawcett, 1984); for the interprofessional team (CNAs, LPNs, MHPs, and physicians), their observations and expectations of rehabilitation nursing contributions; for nurse managers, their common expectations of nursing contributions; and for patients and families, their perceptions of the specific nurse’s role in rehabilitation. Focus groups included six open-ended questions addressing perceptions of the nurse’s role in rehabilitation, interprofessional collaboration, challenges and opportunities for improvement, and patient-family expectations. Examples of questions included: *“In your view, what distinguishes a rehabilitation nurse from nurses in other specialties?”; “What would you consider to be the ideal posture or stance of a rehabilitation nurse?”; and “Which professional values are most important to you when it comes to supporting patients and their families?”* Master’s students, actively involved in the study design and trained in qualitative research methods, served as facilitators within the focus groups (two in each group). Two nurse directors co-facilitated the patient-family focus group, offering additional clinical insight and fostering a comfortable environment for participants. This dual facilitation approach maintained a patient-centered focus, particularly emphasizing the patient’s perception of the nursing role (Krueger & Casey, 2015).

### Data Analysis

Focus group analyses were conducted using a qualitative method described by Taylor et al. (2018), ensuring a structured and comprehensive six-stage thematic analysis to identify patterns and themes across professional groups, providing an in-depth understanding of how the rehabilitation nursing role is perceived. Master’s students transcribed and reviewed the interview to gain an in-depth understanding of the data. They independently assigned codes to significant data segments, then refined these codes to ensure consistency. Codes were first organized into sub-themes, which were then consolidated into overarching themes, evaluated for coherence, and finalized with clear definitions that reflected their core meaning. Finally, the Ishikawa method was used to organize these themes into actionable insights, guiding the development of targeted project strategies. Given the contextual differences between the two units, data from each unit were analyzed separately to capture unit-specific perspectives and to avoid masking potential variations when aggregating the data. Methodological rigor was ensured through triangulation across diverse participant groups (credibility), independent coding by multiple researchers (dependability), member checking (confirmability), and data saturation (adequate depth and breadth), following established criteria for trustworthiness (Taylor et al., 2018; Braun & Clarke, 2019).

### Methodological Considerations

Although our study included both qualitative and quantitative components, we classified the design as multi-method rather than mixed-methods. At the design stage, the two strands were intended to address different research questions: the quantitative questionnaire aimed to assess how nurses viewed their role in rehabilitation in terms of importance, feasibility, and self-assessed competence, while the qualitative focus groups aimed to explore how nurses, other health care professionals, patients, and families perceived the rehabilitation nursing role within the interprofessional context. The aim was not to investigate the same phenomenon through two paradigms simultaneously, as in a formal mixed-methods design. Nevertheless, some overlap emerged in the analyses, allowing us to highlight convergences and divergences between nurses’ self-assessment (quantitative questionnaire) and external perceptions (focus groups). While this secondary comparison yielded valuable insights, it was not planned a priori and did not involve an explicit integration strategy. Therefore, in line with Creswell and Plano Clark (2017), we consider our design to be multi-method rather than mixed-methods.

### Ethical Issues Related to Human Subjects

This study was conducted in accordance with Swiss legal requirements, was not subject to the Swiss law on medical research involving human subjects (ORH, Art. 2;2013), and adhered to the ethical principles outlined in the Declaration of Helsinki. The research protocol was reviewed and approved by the hospital’s and rehabilitation department’s management. All participants provided oral informed consent for audio recording during the focus groups and for data collection through written questionnaires. Written consent was not required, as the study did not require ethical review under current legislation. No personal or sensitive data were included in the transcripts, and all audio recordings were destroyed after transcription and anonymization.

## Results

### Quantitative Analysis

#### Nurse Characteristics and Questionnaire Uptake

A total of 35 nurses completed the questionnaire, corresponding to a response rate of 46% (17/37) in Unit 1 and 41% (18/44) in Unit 2. Among participants, 40% (14/35) were between 41 and 50 years old, and 20/35 (57.1%) held a bachelor’s degree as their highest level of education. The median professional nursing experience was 15 years (IQR: 7–23.5). In addition, 16/35 (45.7%) nurses reported more than 5 years of experience in rehabilitation nursing and were classified as nurse experts in this field (Benner,1984). No significant socio-demographic differences were observed between participants from Unit 1 and Unit 2 (Table 1).

**Table 1:** Socio-professional Characteristics of Nurses Included in the Quantitative Analysis.

	Overall (*n*=35)	Unit 1 (*n*=17)	Unit 2 (*n*=18)	*p*
Age (years), *n* (%)				0.356
20–30	6 (17.1)	3 (17.6)	3 (17.6)	
31–40	7 (20.0)	2 (11.8)	5 (27.8)	
41–50	14 (40.0)	6 (35.3)	8 (44.4)	
51–60	8 (22.9)	6 (35.3)	2 (11.1)	
Professional nursing experience (year), median (IQR)				0.116
	15 (7–23.5)	22 (12–24)	10 (5.5–20.75)	
Rehabilitation nursing experience^a^, *n* (%)				0.223
Novice (0–2 years)​​​​​	14 (40.0)	6 (35.3)	8 (44.4)	
Intermediate (3–5 years)	5 (14.3)	1 (5.9)	4 (22.2)	
Expert (>5 years)	16 (45.7)	10 (58.8)	6 (33.3)	
Education level, n (%)				0.611
Bachelor’s degree	20 (57.1)	9 (52.9)	11 (61.1)	
Post-graduate training	14 (40.0)	8 (47.1)	6 (33.3)	
Master’s degree	1 (2.9)	0 (0)	1 (5.6)	

Data are presented as median (interquartile range) or as number of cases (%).

Statistical comparisons were performed using Fisher’s exact test for categorical variables and the Wilcoxon rank-sum test for continuous variables.

^a^ Categories were defined as previously described by Benner et al. (1984).

#### Variability of Nurses’ Perceptions Across Domains: Health Promotion, Interprofessional Care, Nursing Interventions, Nursing Role

We examined nurses’ perceptions of their role in rehabilitation across four domains: health promotion, interprofessional care, nurse intervention, and nursing role (Supplemental Table 2, Supplemental Digital Content 1, http://links.lww.com/RNJ/A64). To explore differences among these domains, we compared questionnaire scores based on importance, competence, and feasibility. Overall, we observed significant differences across all three dimensions (importance, *p*<.001; competence, *p*=.003; feasibility, *p*=.03). Specifically, nurses attributed greater importance to health promotion and the nursing role compared with interprofessional care (median 3.75 vs. 3.33, adjusted *p*-value (*p-adj*) =.001 and median 3.67 vs. 3.33, *p-adj*=.001, respectively). Similarly, they considered themselves more competent in health promotion and their nursing role than in interprofessional care (median 3.5 vs. 3, *p-adj*=.003 and median 3.33 vs. 3, *p-adj*=.015, respectively). While similar trends were observed for feasibility, these comparisons did not reach statistical significance (health promotion >interprofessional care, *p-adj*=.059; nursing role >interprofessional care, *p-adj*=.085) (Figure 2A).

**Figure 2:**
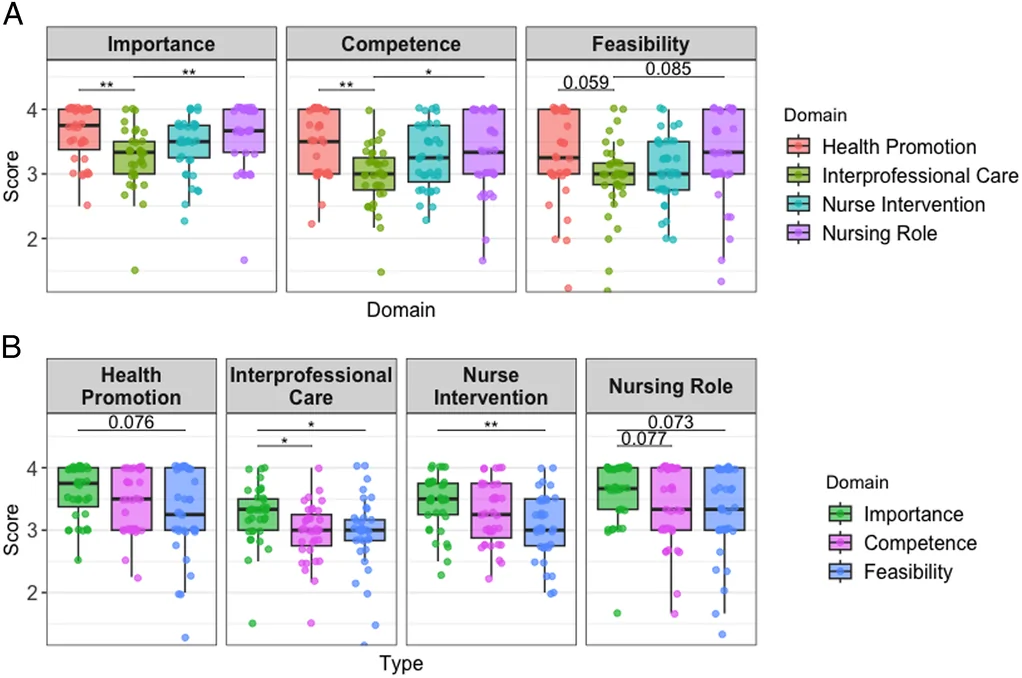
Quantitative comparison of domain-level and type-level nurse questionnaire scores. Median scores for each domain per type (A) and each type per domain (B). Each dot represents the score for an individual participant for a given domain and type, calculated as the average of the scores for the corresponding questionnaire items. Statistical comparisons were performed using the Kruskal-Wallis test, followed by Dunn’s post hoc test with a correction for multiple comparisons. Significance levels are indicated as follows: ***p-adj ≤.001, **p-adj ≤.01, and *p-adj ≤.05. Absolute *p-adj* values are shown for statistical trends (.05 <*p-adj* ≤.1).

Next, we repeated these comparisons separately for each unit to assess potential unit-specific patterns (Supplemental Table 3, Supplemental Digital Content 1, http://links.lww.com/RNJ/A64). Consistent with the findings in the combined group, scores for health promotion and nursing role were higher than those for interprofessional care in both Unit 1 and Unit 2. However, these differences did not always reach statistical significance, likely due to the limited sample size in subgroup analyses (Unit 1: importance, *p*=.071; competence, *p*=.036; feasibility, *p*=.080; Unit 2: importance, *p*=.009; competence, *p*=.140; feasibility, *p*=.4). Despite this, we observed a strong statistical difference in the domains nurses in Unit 2 considered most important (*p*=.009), specifically reflecting a higher perceived importance of health promotion and the nursing role over interprofessional care (median 3.75 vs. 3.33, *p-adj*=.016 and median 3.67 vs. 3.33, *p-adj*=.026, respectively).

#### Relationships Between Importance, Competence, and Feasibility by Domain

To further examine nurses’ perceptions on their role in rehabilitation, we compared the relationships between importance, competence, and feasibility within each domain (Supplemental Table 2, Supplemental Digital Content 1, http://links.lww.com/RNJ/A64). We observed strong differences in interprofessional care (*p*=.006), which nurses perceived as more important than their competence in it (median 3.33 vs. 3, *p-adj*=.01) and more important than its feasibility (median 3.33 vs. 3, *p-adj*=.016). A similar pattern was observed in the nursing role, although the differences were at the threshold of statistical significance (*p*=.05). In addition, nurse intervention showed significant overall differences (*p*=.012), specifically perceived as more important than feasible (*p-adj*=.009). A similar trend was noted in health promotion (*p*=.076), but the difference between importance and feasibility did not reach statistical significance (*p-adj*=.076) (Figure 2B).

We then performed these comparisons at the unit level (Supplemental Table 3, Supplemental Digital Content 1, http://links.lww.com/RNJ/A64). While no statistically significant differences were found in Unit 1, we observed differences in interprofessional care (*p*=0.022) and the nursing role (*p*=0.041) in Unit 2, with both domains being rated as more important than feasible (median 3.33 vs. 3, *p-adj*=0.025 and median 3.67 vs. 3.17, *p-adj*=0.05). A similar trend of higher importance than competence was noted in these domains, though these differences did not reach statistical significance (*p-adj*=.065 and *p-adj*=.086). Finally, health promotion showed a trend toward being perceived as more important than feasible, but this too did not reach statistical significance (*p-adj*=.074).

### Qualitative Analysis

#### Participant Characteristics

In Unit 1, we enrolled six nurses, six CNAs/LPNs, three MHPs, and two physicians. The average professional experience was 7.6, 17.5, and 11.6 years for nurses, CNAs/LPNs, and MHPs respectively; this information was not collected for the physician group. Regarding rehabilitation experience, the averages were 6.2, 10.4, 4.7, and 3.75 years for the four respective groups. In Unit 2, we enrolled four nurses, four CNAs/LPNs, three MHPs, and three physicians, with an average professional experience of 4.0, 18.8, 12.3 and 8.5 years, and an average experience in rehabilitation of 2.9, 5.0, and 4.2 years, respectively. Across both units, there were also five nurse managers, one dietitian manager, one physiotherapist manager and one occupational therapist manager. In addition to health care professionals, we enrolled six patients, whose hospitalization period ranged from 1 week to 1 month, and six patient relatives, whose family members were hospitalized for 2 weeks to 6 months (Figure 1).

#### Themes

The analysis of focus groups highlighted two main themes, which were common between Unit 1 and Unit 2:

Theme 1: Unclear boundaries of the specific nursing role in rehabilitation. In both units, nurses emphasized their role in promoting patient autonomy and supporting patients in transitioning to the next stage of care, whether returning home or continuing their recovery process:

*“What I seek to promote is a certain degree of autonomy for the patient, having a sufficient level of health that allows them to go home or move to the next stage.”*

Another nurse highlighted the importance of partnership, stating,

*“The idea in rehabilitation, what’s interesting, is to consider the patient/family as partners.”*

However, the level of awareness differed depending on the context of each unit. In Unit 1, the caring philosophy aligned more naturally with the nursing identity, while in Unit 2, nurses perceived their role as being in transition between acute care and rehabilitation care. Despite this awareness, nurses in both units reported that their role remains only partially deployed and often unclear, both for themselves and for the interprofessional team.

*“We have no definition of nursing objectives, expected outcomes. We are too medically delegated for most of the care, and then we work with physiotherapists, occupational therapists, etc.”*

Nurses frequently cited organizational barriers, such as lack of time, insufficient support for families, and limited therapeutic education. In addition, they identified institutional challenges, including staff shortages and rigid procedures that enforce patient classification, conflicting with the principles of caring philosophy and humanizing care. Furthermore, nurses expressed frustration with health care insurance providers, whose reimbursement models fail to recognize their specific competencies required in rehabilitation nursing.

*“As expert nurses, our time is not measured or accounted for based on the care provided.”*

In Unit 2, the balance between acute care and rehabilitation was particularly challenging, with nurses feeling insufficiently specialized:

*“So there is reality and practice, and practice really strays away from the patient-partner model,” “We feel ‘half-half’ specialized in acute care and rehabilitation.”*

For CNAs/LPNs, the nurse-CNA/LPN tandem is essential to the rehabilitation process, built on values such as patient-centered care, communication, and trust. CNAs and LPNs contribute to rehabilitation through activities of daily living and promoting autonomy. Yet, they shared the nurses’ concerns, noting that time constraints hinder their ability to fully engage in rehabilitation efforts, particularly given the influx of acute care patients:

*“We put in place small things that are important for rehabilitation, like dressing the patient, trying to walk, or maintaining their autonomy, little things like that.”*

However, they perceive the nursing role as nonspecialized, with a strong emphasis on medically delegated tasks.

*“Rehabilitation is done more with the physiotherapist than with us, so for them (the patients), it’s more the physiotherapist who should work on that than us.”*

Physicians recognized the importance of nurses in the rehabilitation process in both units, but highlighted a lack of nursing leadership and institutional barriers that constrain their role. The nurses they observed often remained tied to medically delegated tasks rather than defining and asserting their unique contributions:

*“The nurse’s own role in our service too often revolves around the doctor and does not inhabit its own roles enough.”*

While collaboration with patients and families is essential to rehabilitation, physicians perceived systemic obstacles such as organizational rigidity and communication issues that limit the full deployment of nursing competencies:

*“The organization itself does not make it easy for the nurse with all these roles. We like to see them execute and have quantifiable actions.”*

MHPs viewed the nursing role in rehabilitation as nonspecialized yet essential, defined by the nurse’s constant presence, holistic vision, and broad scope of action. This includes patient support, nursing care, stimulation, administrative management, coordination, and interprofessional collaboration aimed at a common goal, which is the patient’s discharge plan:

*“They also coordinate many things between the MHP, their care, and the external appointments of our hospital.”*

Despite this recognition, the MHPs acknowledged a lack of clarity in the nursing role. They also pointed to time constraints as a significant barrier that often lead nurses to prioritize tasks for patients rather than working alongside them:

*“Sometimes they will do things a bit simpler because it will take less time.”*

Theme 2: Role misunderstanding and interprofessional collaboration barriers. Implementing care based on the Caring philosophy remains abstract and challenging within the institutional context, as it is hindered by multiple organizational, institutional, and interprofessional barriers. Nurses reported a lack of clarity regarding role distribution within the care team, leading to misunderstandings and inconsistent interpretations of their contributions to rehabilitation. Moreover, nurses reported rigid institutional procedures often contradict of Caring philosophy, making it difficult to align care with patient and family needs:

*“We are taught Humanitude, Caring, to personalize care, and then we encounter guidelines that do the opposite.”*

The LPNs/CNAs emphasized the inseparable partnership between themselves and nurses, a collaboration rooted in the values of the caring philosophy. This tandem relationship is seen as central to ensuring continuity and quality of care:

*“The complicity remains between the nursing assistant and the nurse,” shared one participant, while another described it as “a role of collaboration among everyone.”*

However, MHPs and physicians observed that interprofessional collaboration with nurses remains suboptimal. They reported challenges along with unclear definitions of the nurses’ roles within the interprofessional team:

One MHP explained, *“They have the guidelines, mobilization protocols, and activity schedules, including getting patients out of bed. That’s the key aspect, it’s a mindset. However, in practice, they focus more on technical procedures than on patient mobilization.”*

Physicians also noted that historical beliefs within the hospital environment affect the health care team’s perceptions of the nursing role in rehabilitation:

*“The weight of history, the perspective each of us has on the nurse colleagues, also greatly influences their place. (…) probably, many doctors and the hospital were built with these questions of influence, of power.”*

For MHPs too, the nursing role in the rehabilitation process remains somewhat unclear. Nurses are recognized for their continuous presence, their relational, technical, and coordination skills. However, their role in physical rehabilitation is less prominent, inhibiting effective collaboration with MHPs:

*“It’s part of a process that has been going on for a very long time (…) each specialist had their own perspective, the occupational therapist as well, and the caregivers were somewhat left aside,”* shared one physiotherapist.

Meanwhile, patients and families viewed the nursing role as primarily centered on providing psychological support, with physical recovery being the responsibility of occupational therapists and physiotherapists:

*“What should the nurse do to help with your rehabilitation? Everything that can promote the human aspect, relationships, and communication are the most important points to develop.”*

Nurse managers described the nurse-patient relationship as being built on relational skills, coordination, feedback, and thorough clinical and social evaluations. They highlighted the importance of clear communication between health care team members and shared interprofessional goals to support rehabilitation objectives:

*“Being able to communicate clearly with common interprofessional goals on rehabilitation objectives” is essential, noted one nurse manager.”*

Across all perspectives, the nurse emerges as a central reference figure for the interprofessional team, the patient, and their families, playing a pivotal role in integrating therapeutic domains:

*“The nurse has one of the most important roles in the rehabilitation process,”* said a nurse manager, while a physician added, *“They are the ones who integrate all the therapy domains, whether it’s physio, occupational therapy, speech therapy, and us too.”*

## Discussion

In this multi-method study, we found that although the role of nurses in rehabilitation is widely acknowledged, their specific contributions often remain unclear to both interprofessional teams and patients or families. This lack of role clarity hinders effective collaboration and limits the full deployment of nursing skills, with potential implications for patient outcomes. Quantitatively, nurses consistently recognized the importance of their role but reported lower competence and feasibility, particularly in the context of interprofessional care. Qualitative findings contextualized this discrepancy, showing how misperceptions of the nursing role, identity ambiguity, and organizational barriers reduce nurses’ ability to enact their role fully.

These findings align with previous studies in the literature. Through a systematic review and meta-ethnography, Clarke et al. (2014) highlighted that nurses play a vital yet often underestimated role in stroke rehabilitation, facing blurred role boundaries and limited involvement in decision-making processes. Similarly, Shirozhan et al. (2022), in a qualitative study conducted in a rehabilitation hospital, identified key barriers to effective rehabilitation nursing care, such as insufficient training, unclear responsibilities, and poor interdisciplinary collaboration. Digby et al. (2018) and Loft et al. (2017) likewise reported persistent uncertainties around nursing roles in interprofessional settings despite the importance of clear role definitions for effective collaboration and improved patient outcomes. Taken together, these studies demonstrate that the quantitative gap between perceived importance and feasibility is explained by qualitative accounts of insufficient preparation, role misperceptions, structural challenges, opening perspectives for implementation and clinical recommendations, detailed below.

Our qualitative data further highlight that nurses see themselves as central to promoting patient autonomy and facilitating transitions to new health statuses, expecting to take more action in their rehabilitation role. However, they also describe an unclear vision of their professional identity and cite various organizational and institutional barriers that constrain their practice, such as a lack of time, insufficient support for families, limited therapeutic education, staff shortages, rigid procedures that enforce patient classification, and conflicts with the principles of caring philosophy and humanizing care. Developing a professional identity specific to rehabilitation nursing requires a combination of technical expertise, interprofessional role positioning, relational care, and interprofessional collaboration. It also relies on reflective processes that enhance visibility, strengthen clinical leadership, and assert nursing practice both within interprofessional teams and in interactions with patients and families (Joseph et al., 2021; Rasmussen et al., 2018; Kilpatrick et al., 2020).

While the limited sample size prevents definitive conclusions, our quantitative findings suggest potential differences between the two units, despite the convergence of the themes identified in the qualitative part. In Unit 2, where nurses and other health care professionals manage both acute care and rehabilitation, interprofessional care is perceived as more difficult to implement. The observed gaps between perceived importance and feasibility, as well as between importance and self-assessed competence in interprofessional care and nursing roles, highlight the potential need for additional training or clinical experience. In contrast, Unit 1, which is focused on and grounded in a caring philosophy, places greater emphasis on relational and holistic care. This contrasts with the broader and more diverse demands of Unit 2, which cares for two distinct patient populations: those requiring acute medical care and those undergoing rehabilitation. The convergence of qualitative themes across units suggests shared challenges in role clarity, while their divergence highlights how organizational culture and care context shape perceptions differently. These complementary analyses revealed both common barriers and unit-specific influences on nurses’ confidence and role enactment, providing a more nuanced understanding of the nursing role in rehabilitation.

### Strengths and Limitations of the Study

Our study has some limitations. First, the relatively small sample size within each professional group has limited the range of statistically feasible quantitative comparisons and subgroup analyses, and potentially, the diversity of themes identified within each group. Nevertheless, the overall qualitative dataset was substantial; conducting 11 focus groups with a total of 50 participants generated rich and varied discussions, and the themes demonstrated high consistency across individuals, supporting the credibility and clinical relevance of the findings. Although the study was conducted at two very different sites, the limited collection of socio-professional characteristics may restrict the generalizability to other contexts. However, our findings provide a valuable foundation for future research involving larger and more diverse participant groups. Lastly, convenience sampling was employed, with health professionals recruited based on their availability on the days of the focus group sessions, while patients and families were approached through a partnering association. This approach may have introduced selection bias.

Despite these limitations, our study provides valuable insights into perceptions of the nursing role in rehabilitation and its impact on interprofessional collaboration. Combining multiple focus groups with a structured focus group guide enabled consistent theme identification and ensured comparability across groups, thereby enhancing the validity of the results. Importantly, the meaningful participation of patients and families contributed to a more nuanced and comprehensive understanding of the perspectives explored. Overall, the complementary use of quantitative and qualitative approaches allowed each method to reinforce the findings of the other, thereby strengthening the study’s conclusions.

## Recommendations for Clinical Practice

This study highlights the need to clarify and strengthen the role of rehabilitation nurses within interprofessional teams. The discrepancy between nurses’ high perceived importance of their role and their low perceived competence and feasibility, particularly in interprofessional care, reflected misperceptions and organizational barriers, which could be effectively addressed by structured frameworks and nursing theoretical models (Kirkevold, 2010; Routasalo et al., 2004; Vaughn et al., 2016). By combining self-reflection with continuous professional development, such models may facilitate the construction and evolution of professional identity within rehabilitation practice (Joseph et al., 2021; Rasmussen et al., 2018).

A well-balanced health care team must recognize each professional’s unique role to optimize patient care (Clarke, 2014; Routasalo et al., 2004; Vaughn et al., 2016). However, the lack of a clear role definition may hinder rehabilitation nursing from establishing itself as a distinct specialty. Effective professionalization depends on specialized knowledge and institutional recognition of nurses’ unique skills (Shirozhan et al., 2022). Without this clarity, rehabilitation care may be perceived as fragmented rather than as a cohesive and distinct discipline (Clarke, 2014; Shirozhan et al., 2022). To address this, implementing targeted training and team-building programs (such as simulations and debriefing sessions), specialized certifications, and supportive policies is recommended to enhance role clarity and strengthen professional recognition in rehabilitation nursing (Kilpatrick et al., 2020). Supportive policies may include institutional frameworks that formally integrate rehabilitation nursing roles into care pathways, policies that allocate protected time for interprofessional collaboration, and organizational incentives for advanced training and certification. At the system level, national or regional policy recognition of rehabilitation nursing as a specialty could further consolidate its status and visibility. These strategies align with the Canadian Interprofessional Health Collaborative’s (2010) competency domains, particularly “role clarification” and “team functioning.”

Finally, our findings underscore the importance of leadership: nurses who felt more confident in their role were also more engaged in collaboration. Strong clinical leadership has been linked in prior studies to improved collaboration and outcomes (Abdulrahman et al., 2022; Digby et al., 2018). By developing clinical expertise, interpersonal understanding, communication, collaboration, and coordination, the five key attributes of clinical leadership identified by Patrick et al. (2011), nurses can strengthen their role within rehabilitation teams. Our findings suggest that actively embodying these competencies in daily practice may be a particularly effective strategy for reinforcing nursing leadership capacity.

## Conclusion

This study highlights the uncertainty about the role of rehabilitation nurses within interprofessional teams and emphasizes the importance of clearly defining roles to improve collaboration and patient outcomes. Using a multi-method approach, our findings revealed that while rehabilitation nurses contribute significantly to patient care, their specific roles often remain unclear not only to colleagues and patients but also to nurses themselves. This ambiguity may hinder collaboration and restrict optimal utilization of nursing skills. Developing a clear professional identity is essential within today’s increasingly complex health care environment. Integrating nursing theoretical models and ongoing professional development can further highlight and reinforce the unique contributions of nursing care in rehabilitation.

Future research should focus on integrating role clarification and leadership development into nursing education and the onboarding processes for new nurses entering rehabilitation services. Adopting this proactive approach early in nurses’ careers will better prepare them to embrace their specialized roles, ensuring smoother transitions into practice and more effective interprofessional collaboration. By instilling these skills from the outset, nurses will be better equipped to navigate the complexities of rehabilitation care, ultimately ensuring higher quality care for patients.

Key Practice Points

The appropriation of the specific rehabilitation role by nurses within an interprofessional team and in collaboration with the patient can be structured into three steps:

- Clarification of the role within the nursing team through evidence-guided reflective sessions, as nurses reported uncertainty about role boundaries.
- Clarification of the role in interprofessional practice through structured simulation-based training, addressing difficulties other professionals had in distinguishing nursing contributions.
- Promotion of clinical nursing leadership through additional evidence-guided reflective sessions, given that both health care professionals noted a lack of nursing leadership and patients/families emphasized the importance of nurses as coordinators of care.

## Supplementary Material
