# Supplementary material for: Clarifying the Specific Role of Nurses in Rehabilitation is Key to Enhancing Interprofessional Collaboration: A Multi-method Study
Source: Rehabil Nurs. 2026 Apr 9;51(4):160–72. doi: 10.1097/RNJ.0000000000000538 (PMC13716296; doi:10.1097/RNJ.0000000000000538)
Supplement: Supplementary file 1 [file rnj-51-160-s001.docx]

**Supplementary Table 1. Nurse Questionnaire**

**NURSE QUESTIONNAIRE**

| **Sociodemographic Data** | |
| --- | --- |
| Age | 20 - 30 years  31 - 40 years  41 - 50 years  51 - 60 years  61 years and more |
| **Socioprofessional Data** | |
| Current Position: |  |
| I have been a nurse for: | _______ month(s) or _______ year(s) |
| I have been working in the unit for: | 0 to 2 years  3 to 5 years  More than 5 years |
| Postgraduate Training : | Certification / Licence  Master’s Degree |

| **1: Not important/competent/feasible at all 2: Slightly important/competent/feasible 3: Important/competent/feasible 4: Very important/competent/feasible** | | **Why is it important for you to implement this?** | | | | **Why do you feel competent to do this?** | | | | **Why is it feasible in your unit/organization?** | | | |
| --- | --- | --- | --- | --- | --- | --- | --- | --- | --- | --- | --- | --- | --- |
|  |  | **1** | **2** | **3** | **4** | **1** | **2** | **3** | **4** | **1** | **2** | **3** | **4** |
| **Nurse Interventions** | I plan, adapt, and evaluate the effectiveness of support technology (such as the use of a smartphone by the patient to remind them to take medications) to improve client care outcomes. | ○ | ○ | ○ | ○ | ○ | ○ | ○ | ○ | ○ | ○ | ○ | ○ |
|  | I identify gaps in care in order to implement new strategies based on research findings to adjust the rehabilitation plan in collaboration with the patient and their family. | ○ | ○ | ○ | ○ | ○ | ○ | ○ | ○ | ○ | ○ | ○ | ○ |
|  | I develop an individualized education plan, implement it, and adapt it in collaboration with the patient and their family (in relation to disability, chronic illness, and health management). | ○ | ○ | ○ | ○ | ○ | ○ | ○ | ○ | ○ | ○ | ○ | ○ |
|  | I conduct a holistic assessment and identify strengths to develop or adapt the rehabilitation plan, respecting the values and culture of the patient and their family. | ○ | ○ | ○ | ○ | ○ | ○ | ○ | ○ | ○ | ○ | ○ | ○ |
| **Health Promotion and Return to Balance** | I assess the patient to determine their ability and commitment to health promotion. | ○ | ○ | ○ | ○ | ○ | ○ | ○ | ○ | ○ | ○ | ○ | ○ |
|  | I identify the physical and/or psychosocial barriers that hinder the patient's ability to self-manage. | ○ | ○ | ○ | ○ | ○ | ○ | ○ | ○ | ○ | ○ | ○ | ○ |
|  | I identify the barriers that could influence the transition of care and coordinate the necessary resources to ensure a safe transition. | ○ | ○ | ○ | ○ | ○ | ○ | ○ | ○ | ○ | ○ | ○ | ○ |
|  | I coordinate the necessary resources to ensure a safe transition. | ○ | ○ | ○ | ○ | ○ | ○ | ○ | ○ | ○ | ○ | ○ | ○ |
| **Nursing role** | I identify quality indicators (pressure ulcers, falls, satisfaction, etc.) and assist in collecting them. | ○ | ○ | ○ | ○ | ○ | ○ | ○ | ○ | ○ | ○ | ○ | ○ |
|  | I use best practice recommendations to develop innovative strategies to improve practice. | ○ | ○ | ○ | ○ | ○ | ○ | ○ | ○ | ○ | ○ | ○ | ○ |
|  | I share strategies derived from best practice recommendations with the team. | ○ | ○ | ○ | ○ | ○ | ○ | ○ | ○ | ○ | ○ | ○ | ○ |
|  | I have an in-depth knowledge of cantonal and federal health policy regarding rehabilitation nursing practice. | ○ | ○ | ○ | ○ | ○ | ○ | ○ | ○ | ○ | ○ | ○ | ○ |
|  | I engage politically for the advancement of the profession (member of the Swiss Nurses Association, member of a political party, institutional think tank, etc.). | ○ | ○ | ○ | ○ | ○ | ○ | ○ | ○ | ○ | ○ | ○ | ○ |
|  | I empower the patient and their family to make shared decisions and use conflict resolution strategies when necessary to mediate disagreements. | ○ | ○ | ○ | ○ | ○ | ○ | ○ | ○ | ○ | ○ | ○ | ○ |
| **Interprofessional Care** | I help coordinate the rehabilitation plan by maximizing the contribution of each member of the interprofessional team during meetings (e.g., CIR meeting). | ○ | ○ | ○ | ○ | ○ | ○ | ○ | ○ | ○ | ○ | ○ | ○ |
|  | I collaborate with the interprofessional team to establish and evaluate a client-centered rehabilitation plan using nursing practice strategies based on best practice recommendations. | ○ | ○ | ○ | ○ | ○ | ○ | ○ | ○ | ○ | ○ | ○ | ○ |
|  | I play an active role in collaboration with the interprofessional team regarding the objectives and priorities of the rehabilitation plan and serve, if necessary, as a mediator in discussions in case of conflict between the team and the patient. | ○ | ○ | ○ | ○ | ○ | ○ | ○ | ○ | ○ | ○ | ○ | ○ |
| **Philosophy of Care** | What is the philosophy of care that guides my professional practice? |  | | | | | | | | | | | |
|  | What is the philosophy of care applied by the institution? |  | | | | | | | | | | | |
|  | Do I adhere to this institutional philosophy? |  | | | | | | | | | | | |

Adapted from Vaughn, S., Mauk, K. L., Jacelon, C. S., Larsen, P. D., Rye, J., Wintersgill, W., Cave, C. E., & Dufresne, D. (2016). The Competency Model for Professional Rehabilitation Nursing. *Rehabilitation Nursing: The Official Journal of the Association of Rehabilitation Nurses, 41(1), 33-44.* <https://doi.org/10.1002/rnj.225>

# Supplementary Table 2. Nurse questionnaire perception scores (Likert scale 1–4) by domain.

| Domain | Type | Median (IQR) |
| --- | --- | --- |
| Health promotion | Importance | 3.75 (3.38-4) |
| Health promotion | Competence | 3.5 (3-4) |
| Health promotion | Feasibility | 3.25 (3-4) |
|  | | |
| Interprofessional care | Importance | 3.33 (3-3.5) |
| Interprofessional care | Competence | 3.0 (2.75 - 3.25) |
| Interprofessional care | Feasibility | 3.0 (2.83 - 3.17) |
|  | | |
| Nurse intervention | Importance | 3.5 (3.25 - 3.75) |
| Nurse intervention | Competence | 3.25 (2.88 - 3.75) |
| Nurse intervention | Feasibility | 3.0 (2.75 - 3.5) |
|  | | |
| Nursing role | Importance | 3.67 (3.33 – 4) |
| Nursing role | Competence | 3.33 (3 – 4) |
| Nursing role | Feasibility | 3.33 (3 – 4) |

Median scores (with interquartile ranges, IQR) from the Nurse Questionnaire assessing nurses’ perceptions of their role across four domains (*Health promotion, Interprofessional care, Nurse interventions,* and *Nursing role*). Each domain was evaluated in terms of perceived importance, self-assessed competence, and perceived feasibility on a 4-point Likert scale (1 = lowest, 4 = highest).

**Supplementary Table 3. Nurse questionnaire perception scores (Likert scale 1–4) by domain for each site.**

| Domain | Type | Site | Median (IQR) |
| --- | --- | --- | --- |
| Health Promotion | Importance | Unit 1 | 3.5 (3.25 – 4) |
| Health Promotion | Importance | Unit 2 | 3.75 (3.5 – 4) |
| Health Promotion | Competence | Unit 1 | 3.5 (3 – 4) |
| Health Promotion | Competence | Unit 2 | 3.5 (3 - 3.94) |
| Health Promotion | Feasibility | Unit 1 | 3.25 (3 – 4) |
| Health Promotion | Feasibility | Unit 2 | 3.12 (2.81 - 3.94) |
|  |  |  |  |
| Interprofessional Care | Importance | Unit 1 | 3.17 (3 - 3.5) |
| Interprofessional Care | Importance | Unit 2 | 3.33 (3.17 - 3.46) |
| Interprofessional Care | Competence | Unit 1 | 3.0 (2.67 - 3.17) |
| Interprofessional Care | Competence | Unit 2 | 3.0 (2.83 - 3.29) |
| Interprofessional Care | Feasibility | Unit 1 | 3.0 (2.83 - 3.17) |
| Interprofessional Care | Feasibility | Unit 2 | 3.0 (2.71 - 3.12) |
|  |  |  |  |
| Nurse Intervention | Importance | Unit 1 | 3.5 (3.25 - 3.75) |
| Nurse Intervention | Importance | Unit 2 | 3.5 (3.25 - 3.75) |
| Nurse Intervention | Competence | Unit 1 | 3.25 (3 - 3.75) |
| Nurse Intervention | Competence | Unit 2 | 3.12 (2.75 - 3.5) |
| Nurse Intervention | Feasibility | Unit 1 | 3.0 (2.75 - 3.5) |
| Nurse Intervention | Feasibility | Unit 2 | 3.0 (2.75 - 3.5) |
|  |  |  |  |
| Nursing Role | Importance | Unit 1 | 4.0 (3.33 – 4) |
| Nursing Role | Importance | Unit 2 | 3.67 (3.42 – 4) |
| Nursing Role | Competence | Unit 1 | 3.67 (3 – 4) |
| Nursing Role | Competence | Unit 2 | 3.33 (2.75 - 3.92) |
| Nursing Role | Feasibility | Unit 1 | 3.67 (3 – 4) |
| Nursing Role | Feasibility | Unit 2 | 3.17 (3 - 3.67) |

Median scores (with interquartile ranges, IQR) from the Nurse Questionnaire assessing nurses’ perceptions of their role across four domains (*Health promotion, Interprofessional care, Nurse interventions,* and *Nursing role*) for each site (unit 1 & unit 2). Each domain was evaluated in terms of perceived importance, self-assessed competence, and perceived feasibility on a 4-point Likert scale (1 = lowest, 4 = highest).
